# Physical and Mechanical Evaluation of Five Suture Materials on Three Knot Configurations: An *in Vitro* Study

**DOI:** 10.3390/polym8040147

**Published:** 2016-04-20

**Authors:** Desire Abellán, José Nart, Andrés Pascual, Robert E. Cohen, Javier D. Sanz-Moliner

**Affiliations:** 1 Department of Periodontics, School of Dentistry, Universitat Internacional de Catalunya (UIC), Barcelona 08195, Spain; abellan.d@uic.es (D.A.); josenart@hotmail.com (J.N.); pascuallarocca@hotmail.com (A.P.); 2 Department of Periodontics and Endodontics, University at Buffalo, Buffalo 14260, NY, USA; rcohen@buffalo.edu

**Keywords:** suture materials, failure load, physical conditions, knot configuration, knot slippage

## Abstract

The aim of this study was to evaluate and compare the mechanical properties of five suture materials on three knot configurations when subjected to different physical conditions. Five 5-0 (silk, polyamide 6/66, polyglycolic acid, glycolide-e-caprolactone copolymer, polytetrafluoroethylene) suture materials were used. Ten samples per group of each material were used. Three knot configurations were compared A.2=1=1 (forward–forward–reverse), B.2=1=1 (forward–reverse–forward), C.1=2=1 (forward–forward–reverse). Mechanical properties (failure load, elongation, knot slippage/breakage) were measured using a universal testing machine. Samples were immersed in three different pH concentrations (4,7,9) at room temperature for 7 and 14 days. For the thermal cycle process, sutures were immersed in two water tanks at different temperatures (5 and 55 °C). Elongation and failure load were directly dependent on the suture material. Polyglycolic acid followed by glycolide-e-caprolactone copolymer showed the most knot failure load, while polytetrafluoroethylene showed the lowest (*p* < 0.001). Physical conditions had no effect on knot failure load (*p* = 0.494). Statistically significant differences were observed between knot configurations (*p* = 0.008). Additionally, individual assessment of suture material showed statistically significant results for combinations of particular knot configurations. Physical conditions, such as pH concentration and thermal cycle process, have no influence on suture mechanical properties. However, knot failure load depends on the suture material and knot configuration used. Consequently, specific suturing protocols might be recommended to obtain higher results of knot security.

## 1. Introduction

Sutures are used in surgery for a number reasons. These include the reapproximation of tissues separated as a result of surgery or trauma, enhancement of primary healing and hemorrhage control [1]. The most commonly used materials for this purpose are sutures [2].

Sutures play a key role in ligating severed ends of vessels and approximating tissues. The knot is the weakest part of a suture. There are many factors involved in the overall integrity of a knot, such as tension, diameter of the suture, kind of knot, number of throws and length of the cut ends [3].

In periodontal surgery, the appropriate choice of suturing technique, surgical needle, thread diameter, thread type, as well as an adequate surgical knot for each thread material chosen, are all crucial to achieve optimal healing [4]. In the oral cavity, suturing differs from other areas of the body due to the type of tissues involved, the permanent presence of saliva, high tissue vascularization, speech-related functions, mastication, and swallowing. Sutures demand a number of specific characteristics and properties, such as dimensional stability, lack of memory, good tensile strength, knot security, and enough flexibility to prevent damage to the oral mucosa [5].

A range of sutures are available that are classified by several criteria: 1. Composition—natural and synthetic; 2. structure—monofilament and multifilament; and 3. spontaneous degradation—absorbable and non-absorbable [2].

One of the advantages of absorbable sutures is that they generally do not need to be removed. However, those materials are associated with different tissue responses as a result of their degradation by hydrolysis, enzymatic digestion or phagocytosis. The rate or this degradation depends on the pH and the temperature of the tissues surrounding the suture [2].

Periodontists select the type of suture material according to several criteria that include absorbability, handling, strength, and the structure of the suture. Nonetheless, research has shown that monofilament sutures induce less tissue reaction than do multifilament sutures [2,6,7].

Monofilament sutures have lower knot tie-down resistance, lower tissue drag and less risk of infection compared with braided suture materials. There is less risk of colonization by microorganisms and they are easier to tie. However, their cut ends could irritate mucosa and cause ulceration [2].

Multifilament sutures are easy to handle and tie because they have lower bending stiffness and are easy to form a stable knot. However, their braided structure often facilitates the accumulation of food debris or bacteria [2].

Among monofilament sutures, polytetrafluoroethylene (PTFE) has low surface friction, chemical inertness, biocompatibility, resistance to *in vivo* degradation, minimal memory and, consequently, superior handling characteristics. However, the low static coefficient of friction and chemical inertness make the knot less secure [8].

Of the different suture materials, polyglycolic acid (PGA) has a lower incidence of infection in contaminated tissues and exhibits no residual tensile strength [9]. As a result, the PGA is stronger than silk, but its strength lessens significantly after a period of time in the oral tissues. Low-cycle tensile fatigue testing showed that breaking strengths of PGA sutures dropped appreciably by cyclic loading and degradation, with a significant decrease in tensile strength, after a period of five to seven days in the oral tissues [6].

An in-depth understanding of the physical and mechanical properties of suture materials is crucial to dental practice. However, there is incomplete and inconsistent information regarding tissue reactions to different suture materials, above all in humans [7]. At present, there is no suture that meets all the requirements of an ideal material [10]. Hence, the aims of the present study are to evaluate and compare the mechanical properties of five suture materials and three knot configurations when exposed to physical conditions.

## 2. Material and Methods

This *in vitro* study was designed in 2010 and conducted between March 2011 and December 2012. The protocol of the study (PER-ELM-2011-01-CM) was evaluated by the Scientific Committee of the Universitat Internacional de Catalunya, Barcelona, on March 30th 2011, who certified the research protocol was adequate to be developed.

Five suture materials were used in order to assess their properties in three different knot configurations (Table 1).

Five 5-0 (silk, polyamide 6/66 (PV), polyglycolic acid (AP7), glycolide-e-caprolactone copolymer (GC7) and polytetrafluoroethylene (PTFE)) suture materials were used in this study. Three were monofilament and two multifilament. Each group contained ten samples. The entire study was carried out by a single examiner.

### 2.1. Sample Preparation

**Figure 1:**
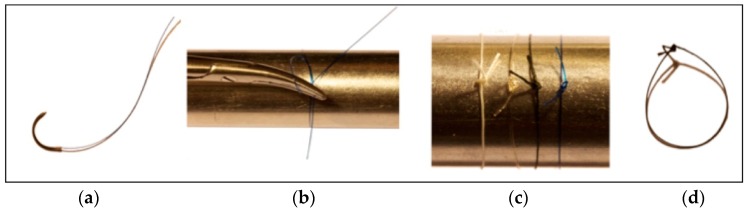
Sample preparation. (**a**) Suture material before tying; (**b**) Tying of suture material around a 26 mm metal cylinder; (**c**) Different suture materials after tying; (**d**) Sample of suture material prepared for testing mechanical and chemical conditions.

Prior to testing, samples were knotted around a 26 mm diameter metal cylinder, leaving a 3 mm knot end length. Three knot configurations for each type of suture material were compared, A.2=1=1 (forward–forward–reverse), B.2=1=1 (forward–reverse–forward), C.1=2=1 (forward–forward–reverse), applying pressure for 3 s after tying each knot (Figure 1).

### 2.2. Mechanical Test

Mechanical properties (failure load, elongation, knot slippage or knot breakage) were measured using a Quasar 5 Universal Testing Machine (UTM) (Galdabini 1890, V925. 2007, Cardano Al Campo, Italy).

Elongation was measured as the displacement that a suture can experience before breaking in tensile testing. The failure load was calculated as the maximum tension the material can withstand without tearing. In addition, knot slippage was defined as a knot that slips easily along the cord or line around which it is made, and knot breakage as the point at which material breaks under loading.

**Figure 2:**
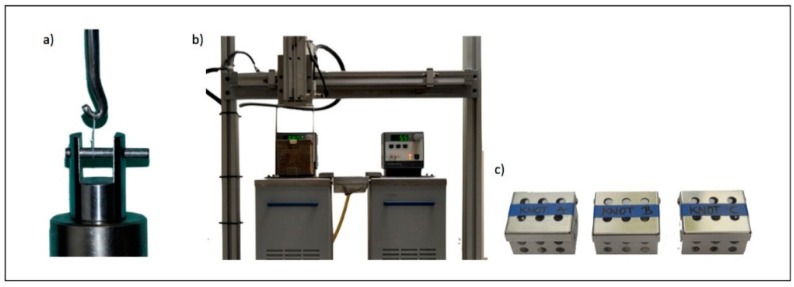
Mechanical and physical tests. (**a**) Hydraulic grip of the Universal Testing Machine; (**b**) 5 and 55 °C water tanks of the Universal Thermal Cycling Testing Machine; (**c**) Metal boxes which contained the sample for the thermal cycle process.

To conduct the analysis, sutures were placed on two hooks at opposite ends of the UTM and tested at 0.25 N/mm at an established mandible separation speed of 50 mm/min (Figure 2a).

### 2.3. Physical Test

Sutures underwent different physical environments. Samples were independently subjected either to pH changes or thermal cycle fluctuations (Figure 2b,c).

Samples tested for pH conditions were immersed in three different flasks at pH = 4, pH = 7 and pH = 9 concentrations. Physical properties were evaluated within 7 and 14 days.

Thermal cycle fluctuations were tested in a Universal Thermal Cycling Testing Machine (UTCTM) (Universitat Internacional de Catalunya, Barcelona, Spain). Sutures were immersed in two water tanks for 30 s/tank at 5 and 55 °C, respectively to simulate oral conditions and evaluate suture degradation. Sutures were submitted to 182 and 364 cycles, which simulates 1 and 2 weeks clinically, based on an estimate of 10,000 cycles/year for biomaterials in the oral environment [11].

After assessment of their physical properties, the mechanical properties of the sutures were evaluated as described above.

### 2.4. Study Groups

Ten samples of suture material were used per group. This entailed a total sample size of 1350 specimens. Suture materials were analyzed according to three different study groups.

*Control group* contained a total of 150 samples: 10 of each suture (silk, polyamide 6/66, polyglycolic acid, glycolide-e-caprolactone copolymer, and polytetrafluoroethylene). The sutures in this group were tested for failure load, elongation, knot slippage or knot breakage. Thirty samples of suture were used according to each knot configuration.

*pH group* contained a total of 900 samples: 180 samples of each suture (10 samples of silk, 10 samples of polyamide 6/66, 10 samples of polyglycolic acid, 10 samples of glycolide-e-caprolactone copolymer and 10 samples of polytetrafluoroethylene). The samples in this group were subjected to 3 different pH concentrations (4,7,9) at room temperature for 7 and 14 days. Four hundred and fifty sutures were used for each immersion time group. Consequently, each pH group contained 30 samples of each suture. Thereafter, sutures were washed in distilled water five times and tested. Lastly, sutures were tested for mechanical properties of failure load, elongation, knot slippage or knot breakage. Sixty samples per suture were used according to each knot configuration.

*Thermal cycle group* contained a total of 300 samples: 60 samples of each suture (10 samples of silk, 10 samples of polyamide 6/66, 10 samples of polyglycolic acid, 10 samples of glycolide-e-caprolactone copolymer and 10 samples of polytetrafluoroethylene). There were 150 sutures per thermal cycling group. The sutures were exposed to a process of 182 and 364 rounds of thermal testing. Thereafter, the sutures were checked for mechanical properties of failure load, elongation, knot slippage or knot breakage. Sixty samples per suture according to each knot configuration.

### 2.5. Statistical Analysis

The statistical analysis was performed using a standardized sample of 10 sutures per group. The effect of the different physical conditions on the mechanical properties of suture materials was evaluated, as well as the impact of using three different knot configurations on suture materials. The results were analyzed using a multifactorial ANOVA (Table S1), chi-Square test and descriptive analysis with a significance level of 0.05 and 95% confidence intervals.

## 3. Results

### 3.1. Mechanical Test

#### 3.1.1. Analysis of Elongation

**Figure 3:**
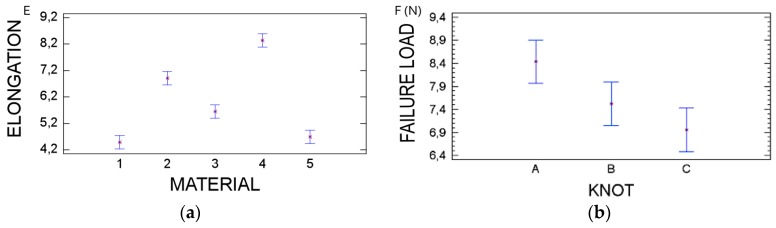
Tables for variables elongation and load failure (**a**) Elongation of suture materials at the three knot configurations; (**b**) Load failure of the suture materials to the different knot configurations.

Elongation at failure varied according to the suture material tested. Greater failure elongations were obtained with PV and GC7, while silk and PTFE exhibited the smallest failure elongations (*p* < 0.001) (Figure 3).

#### 3.1.2. Failure Load Analysis

**Figure 4:**
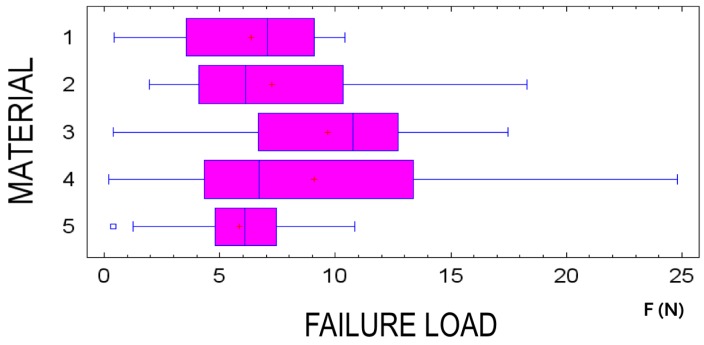
Comparison of suture material failure load in three knot configurations.

Statistical analysis showed that failure load depends on the type of suture material (*p* < 0.001) (Figure 4) and knot configuration used (*p* = 0.008) (Figure 3).

Among the five suture materials evaluated; AP7, GC7, and PV demonstrated the highest values for failure load while Silk and PTFE obtained the lowest results. These findings were statistically significant (*p* < 0.001) (Figure 4).

Moreover, when comparing knot configurations, it was observed that knot configuration A provided more failure load compared to knot configuration B and C. Although, when evaluating all the suture materials in general, this parameter was not found to be statistically significant (*p* = 0.08).

#### 3.1.3. Physical Test

The type of physical condition to which materials were exposed did not affect to any of the parameters that were evaluated in this study. Therefore, physical conditions of pH and temperature seemed not to have an impact on the integrity or failure load of the materials. (*p* = 0.404) (Table 2).

### 3.2. Influence of the Knot Configuration

#### 3.2.1. Knot Failure Load

**Figure 5:**
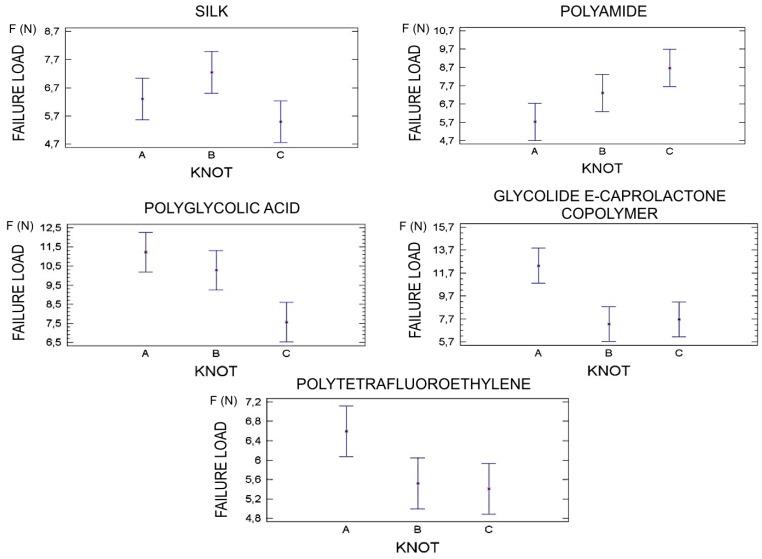
Knot configurations failure load according to the suture material used.

Different failure load patterns were observed depending on the suture material (Figure 4) and knot configuration used (Figure 5).

Silk exhibited higher values in combination with knot configuration B, followed by A and lastly C (*p* = 0.06). PV obtained the best results when knot configuration C was used and the worst in combination with configuration A (*p* = 0.019). AP7 possessed superior failure load with knot configuration A; nonetheless, small differences were found between configurations A and B (*p* = 0.002). GC7 showed a difference between the highest failure load, knot A, and the lowest (*p* = 0.001). However, PTFE showed no difference between knot configurations B and C and exhibited the highest failure load with the use of configuration A (*p* = 0.052) (Figure 5).

An average of the failure load of each suture material in combination with different knot configurations was calculated by using a descriptive analysis. Table 3 shows summary statistics for means and standard deviations.

#### 3.2.2. Knot Breakage or Slippage

**Figure 6:**
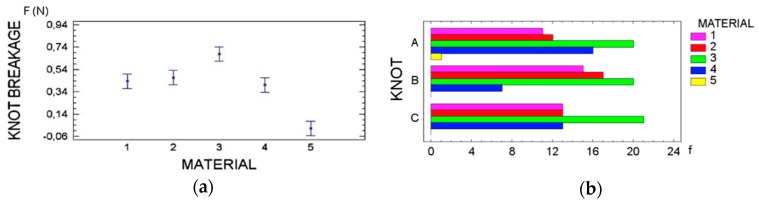
(**a**) Failure load of knots according to each suture material. (**b**) Frequency of knot breakage in relation to suture material and knot configuration used.

Among the five suture materials; AP7 was found to be the most susceptible to knot breakage, followed by PV, Silk and GC7 with few differences between them. Finally, PTFE showed the lowest results in knot breakage but with a high incidence of knot slippage in all the configurations studied (*p* < 0.001) (Figure 6).

## 4. Discussion

This *in vitro* study was designed to evaluate the physical and mechanical properties of 5 suture materials on three knot configurations when exposed to a variety of physical conditions, of which there is scarce information in the literature.

A single examiner carried out the entire study to minimize variability. The methodology used for this research was established according to previous studies. The Universal Testing Machine was configured based on parameters set by Kim *et al.* [2]. Moreover, pH solutions were used in accordance with Chu *et al.* [3] who observed statistically significant differences in the degradation of various suture materials. To date, no study has evaluated the effect of hydration at different temperatures on sutures. However, those criteria have been used as measurement parameters in restorative dentistry, with an increase of microleakage in resin biomaterials [11].

Suture materials used in this study were absorbable or non-absorbable, made of natural or synthetic fibers, and monofilament or multifilament in structure.

Three configurations of three-throw surgeon’s knots were used in this study: knot A with a three-throw forward—one forward and one reverse; knot B with a two-throw forward—one reverse and one forward; and knot C with a one-throw forward two forward and one reverse. The surgeon’s knot was used in this study because it is the most widely used type in periodontal surgery. Moreover, this kind of knot has been previously employed in various studies conducted by Selvig *et al.* [12]. Lekness *et al.* [7] and Yaltirik *et al.* [13]. Suture end length was established at 3 mm according to Muffly *et al.* [14]. Their research demonstrated that 3 mm cut end offers enough length to avoid knot slippage.

Our study demonstrates that suture resistance depends on the type of material and the knot configuration used. Moreover, its physical exposure to biodegradable agents has no effect on suture material integrity or knot resistance. However, other studies evaluating the effects of different media on resorbable suture materials have demonstrated otherwise. A recent report by Fong and coworkers [15] demonstrated that flax fibers sutures that underwent wet conditions exhibited fiber debonding leading to fiber pullout, while dry flax fibers exhibited brittle fracture. However, both wet and dry knotted sutures failed at the entrance of the knot.

Previous studies have reported that absorbable materials have higher resistance to tension while non-absorbable ones show lower resistance. Those results are in agreement with Moser *et al.* [6] in which PGA exhibited the highest results in natural conditions as well as after being immersed in Ringer’s solution from 5 to 7 days. However, that study found a significant decrease in resistance of PGA after remaining from 5 to 7 days in the mouth. In contrast to these results, Lim and coworkers [16] studied the biomechanical properties of a nonabsorbable braided suture, Supramid, with three different suture techniques. The authors concluded that the six-strand single-loop suture technique, pulls out, rather than breaking, at sufficiently high loads and let tissues transfer large forces along the suture. This may suggest that suturing technique influences the degree of load supported.

Some studies have indicated that pH levels may have a greater influence on the performance of absorbable sutures than on non-absorbable ones. Both acidic and alkaline environments are able to accelerate the degradation of absorbable sutures. Among the non-absorbable sutures, silk appears to be the most susceptible to various pH conditions [17]. Silk is the most widely used material despite its suboptimal mechanical properties. This might be due to its easy handling, reliability and strength. In contrast, some studies have concluded that PGA sutures obtained the best strength in an acidic pH condition [18].

## 5. Conclusions

Knot configuration A (three-throw forward—one forward and one reverse) obtained the highest results in knot resistance. This might imply that knot configuration A provides enough resistance and knot security regardless of the configuration used. However, the resistance pattern of the knots varied significantly when different suture materials were used. Silk-based materials obtained more resistant with the use of knot B (two-throw forward—one reverse and one forward); polyamide suture is more resistant with knot C (one-throw forward two forward and one reverse); and the remaining materials analyzed in this study appear to be satisfactory for use with knot configuration A. This may suggest the utilization of standardized protocols for knot configurations may be considered, depending on the type of suture material used.

The suturing protocol obtained from this study may provide higher knot security when the aim is to maintain primary closure in periodontal surgery.

## Figures and Tables

**Table 1:** Five suture materials were used in order to assess their properties.

Suture material	Codification	Composition	Structure	Degradation
Silk ^1^	SILK	Natural	Multifilament	Non absorbable
Polyamide ^2^	PV	Synthetic	Monofilament	Non absorbable
Polyglycolic acid ^3^	AP7	Synthetic	Multifilament	Absorbable
Glycolide-e-caprolactone copolymer ^4^	GC7	Synthetic	Monofilament	Absorbable
Polytetrafluoroethylene ^5^	PTFE	Synthetic	Monofilament	Non absorbable

^1^ Silk. Triangular, 3/8 circle premium DS16 5/0 Ancladén® (Barcelona, Spain). Product code: 5650; ^2^ Polyamide 6/66. PV monofil triangular, 3/8 circle premium DS16 5/0 Ancladén® (Barcelona, Spain). Product code: 7625; ^3^ Polyglycolic acid. AP7 rapid triangular, 3/8 circle premium DS16 5/0 Ancladén® (Barcelona, Spain). Product code: 2604; ^4^ glycolide-e-caprolactone copolymer. GC7 monofil triangular, 3/8 circle Premium DS16 5/0 Ancladén® (Barcelona, Spain). Product code: 4623; ^5^ Polytetrafluoroethylene. PTFE 3/8 circle premium 4/0 Osteogenics® (Lubbock, Texas) Product code: CS 0618.

**Table 2:** Analysis of interaction of variables.

Variables	Interactions	*p*-Value
A: material	AB	<0.001 *
B: knot	CF	0.404
C: group	DA	<0.001 *
D: knot breakage	DB	<0.001 *
E: elongation	AE	<0.001 *
F: failure load	FB	0.008 *
F: failure load	FA	<0.001 *

* Statistically significant.

**Table 3:** Descriptive analysis of sutures failure load at the three knot configurations.

Material	Knot configuration	Analysis of failure load (N)		
		Mean	Variance	Standard deviation
SILK	A	6.298	8.477	2.911
SILK	B	7.242	7.941	2.818
SILK	C	5.494	8.534	2.921
PV	A	5.738	6.435	2.536
PV	B	7.301	18.288	4.276
PV	C	8.665	22.652	4.759
AP7	A	11.218	15.528	3.94
AP7	B	10.276	19.614	4.428
AP7	C	7.557	13.912	3.729
GC7	A	12.342	48.637	6.974
GC7	B	7.252	24.767	4.976
GC7	C	7.668	31.942	5.651
PTFE	A	6.593	2.593	1.61
PTFE	B	5.521	3.55	1.884
PTFE	C	5.41	6.414	2.532

## Supplementary Materials

Supplementary materials can be found at www.mdpi.com/2073-4360/8/4/147/s1.
