# Supplementary material for: Physical and Mechanical Evaluation of Five Suture Materials on Three Knot Configurations: An in Vitro Study
Source: Polymers (Basel). 2016 Apr 20;8(4):147. doi: 10.3390/polym8040147 (PMC6432448; doi:10.3390/polym8040147)
Supplement: Supplementary file 1 [file polymers-08-00147-s001.pdf]

Polymers **2016**, *8*, 147; doi:10.3390/polym8040147

Supplementary Materials: Physical and Mechanical Evaluation of Five Suture Materials on Three Knot Configurations: An *in vitro* Study

Desire Abellán, José Nart, Andrés Pascual, Robert E. Cohen and Javier D. Sanz-Moliner

Table S1. ANOVA Interaction of variables.

<table>
<tr><th>Variables</th><th>Levels</th><th>Interactions</th></tr>
<tr><td rowspan="5">A: material</td><td>Silk</td><td rowspan="3">Material-group</td></tr>
<tr><td>Polyamide</td></tr>
<tr><td>Polyglycolic acid</td></tr>
<tr><td>Glycolide e-caprolactone</td><td rowspan="3">Material-knot</td></tr>
<tr><td>Polytetrafluoroethilene</td></tr>
<tr><td rowspan="3">B: knot</td><td>A. 2=1=1(forward-forward-reverse)</td></tr>
<tr><td>B. 2=1=1 (forward-reverse-forward)</td><td rowspan="3">Material-group-knot-knot breakage</td></tr>
<tr><td>C. 1=2=1 (forward-forward-reverse)</td></tr>
<tr><td rowspan="3">C: group</td><td>control</td></tr>
<tr><td>pH</td><td rowspan="3">Material-group-knot-elongation</td></tr>
<tr><td>thermal cycle</td></tr>
<tr><td>D: knot breakage</td><td>–</td></tr>
<tr><td>E: elongation</td><td>–</td><td rowspan="2">Material-group-knot-failure load</td></tr>
<tr><td>F: failure load</td><td>–</td></tr>
</table>

© 2016 by the authors; licensee MDPI, Basel, Switzerland. This article is an open access article distributed under the terms and conditions of the Creative Commons Attribution (CC-BY) license (http://creativecommons.org/licenses/by/4.0/).
